# *Nigella sativa* stimulates insulin secretion from isolated rat islets and inhibits the digestion and absorption of (CH_2_O)_n_ in the gut

**DOI:** 10.1042/BSR20190723

**Published:** 2019-08-23

**Authors:** J.M.A. Hannan, Prawej Ansari, Afra Haque, Afrina Sanju, Abir Huzaifa, Anisur Rahman, Adity Ghosh, Shofiul Azam

**Affiliations:** 1 Department of Pharmacy, Independent University, Bangladesh (IUB) Bashundhara R/A, Dhaka1229, Bangladesh; 2 School of Biomedical Sciences, Ulster University, Coleraine BT52 1SA, Co. Londonderry, Northern Ireland, United Kingdom; 3 Department of Pharmacy, East West University, Dhaka-1212, Bangladesh; 4 Department of Integrated Bioscience, Graduate School, Konkuk University, Chungju- 7478, Republic of Korea

**Keywords:** Disaccharidase enzyme activity, GI motility, Glucose tolerance, Gut perfusion, Nigella sativa, Sucrose malabsorption

## Abstract

*Nigella sativa* seeds are traditionally reputed as possessing anti-diabetic properties. As a result, we aim to explore the mechanism of its anti-hyperglycemic activity. The present study uses various experimental designs including gastrointestinal (GI) motility, intestinal disaccharidase activity and inhibition of carbohydrate digestion and absorption in the gut. The animals used as type 2 diabetic models were induced with streptozotocin to make them as such. Oral glucose tolerance test was performed to confirm that the animals were indeed diabetic. The extract reduced postprandial glucose, suggesting it interfered with glucose absorption in the gut. It also improved glucose (2.5g/kg, b/w) tolerance in rats. Furthermore, treatment with *N. sativa* produced a significant improvement in GI motility, while reduced disaccharidase enzyme activity in fasted rats. The extract produced a similar effect within an acute oral sucrose (2.5g/kg, b/w) load assay. Following sucrose administration, a substantial amount of unabsorbed sucrose was found in six different parts of the GI tract. This indicates that *N. sativa* has the potentiality to liberate GI content and reduce or delay glucose absorption. A potential hypoglycemic activity of the extract found in insulin release assay, where the extract significantly improved insulin secretion from isolated rat islets. These concluded present findings give rise to the implication that *N. sativa* seeds are generating postprandial anti-hyperglycemic activity within type 2 diabetic animal models via reducing or delaying carbohydrate digestion and absorption in the gut as well as improving insulin secretion in response to the plasma glucose.

## Introduction

Diabetes Mellitus (DM) is a chronic and complex metabolic group of disorders—its prevalence has increased rapidly on a global scale. Mortality rates for DM are estimated to reach a total of 2.9 million deaths by the year 2030. Increasingly, diabetes is cited as a significant global threat to public health [1,2], with 246 million individuals with this polygenic disorder around the world. Eighty percent reside in developing countries [3]. Diabetes is ranked seventh among the leading causes of mortality globally [4].

Type 2 DM is the most common and one of the life-threatening disease conditions among the current classified types. Type 2 DM is usually manifested via obesity and genetic disposition [5]. For the management of DM, the interruption of carbohydrate digestion and absorption is an active therapeutic approach of interest. The presence of aldohexose in the circulation over an extended period combined with the apprehended absorption technique, enables pancreatic β-cells of diabetic individuals to adjust their postprandial metabolic rate [6]. Moreover, the effectiveness of drug therapy is limited, and it shows a variety of complications and side effects [7].

*Nigella sativa* (*N. sativa*), also known as black seed, is a plant which belongs to the family Ranunculaceae and is native to Southern Europe, North Africa, and Southwest Asia [8]. Traditionally black seeds are used to treat a wide range of ailments including different airway disorders, chronic headaches, back pain, diabetes, paralysis, infections, inflammation, hypertension, and digestive tract-related problems. The black seed is administered in different kinds of preparations depending on the ailment. It also has topical uses to treat blisters, nasal abscesses, eczema, and swollen joints [9]. *N. sativa* components exhibit a remarkable array of biochemical, immunological, and pharmacological actions, including bronchodilatory [10], anti-inflammatory [11], antibacterial [12], hypoglycemic [13], and immunomodulatory effects [14]. Most of these properties have been attributed mainly to the quinone constituents of *N. sativa* like thymoquinone (TQ) (30–48%), thymohydroquinone and dithymoquinone (nigellone). Of the quinones, TQ is the most abundant active ingredient of the extracted volatile oils from the black seed [15]. In addition, TQ has been reviewed several times as an antioxidant, anti-inflammatory and anti-tumorigenic [16–18]. TQ has also been reported to reduce hippocampal neurodegeneration following chronic toluene exposure in rats [19] and protects the frontal cortex from similar toxin exposure [20]. Some other studies have reported too that TQs have potentiality to clear Aβ in AD model [21–23].

*N. sativa* has been reported for different anti-diabetic properties in various diabetic animal models, yet no mechanistic investigation has been carried out. Herein, we focused on exposing a more comprehensive mechanism of action of *N. sativa* within diabetic animal models. Previous studies with this extract claim that it may act by improving (or mimicking) insulin secretion or reducing the oxidative stress of β-cells [13,24]. We designed the present study to co-relate gastrointestinal (GI) absorption interference by this extract and observing its anti-diabetic effect as a result. The present study will provide an insight and give a thorough evaluation of the hypoglycemic effects of *N. sativa* in diabetic animal models and will examine the possible effects of *N. sativa* on intestinal glucose absorption and GI motility, as these are part of this plant’s anti-hyperglycemic efficacy.

## Materials and methods

### Plant collection and processing

The seeds of *N. sativa* were purchased from the commercial herbal medicine outlet in Uttara, Dhaka, Bangladesh. Seeds were collected in dried form as ordered and the extract was prepared following the procedure as previously described by Azad et al. [25]. Briefly, fully dried seeds were then ground to make a powder, and 500 g of the powdered material was soaked in 2.5 l of methanol inside a flat-bottomed glass container. The solution was kept for 1 week while being shaken continuously. The mixture after this was first filtered using fresh cotton and at the end it was filtered with filter paper (Whatman no. 1), and the obtained material was evaporated by a Rotary Evaporator (Bibby RE-200, Sterilin Ltd., U.K.) at 5–6 rpm at 57°C. Finally, a gummy, semi-solid crude extract was obtained and stored at 4°C until required in the study.

### Determination of glucose-adsorption capacity

This assay was conducted as described by Ou et al. [26]. In brief, the glucose-adsorption capacity (mmol/l) was measured by mixing 1 g of either insoluble plant powder or carboxymethyl cellulose, with 100 ml of glucose solution. The mixture was incubated at 37°C for 6 h. Afterward, the mixture was centrifuged at 3500 rpm for 15 min. Glucose concentration in the supernatant was assayed using GOD-PAP method as previously described [27].

### Experimental animal models

Long–Evans rats (both male and female), weighing 150–200 g were collected from icddr, b, and acclimatized and bred within the animal house of the Department of Pharmacy, East West University, Dhaka, Bangladesh. Animals were kept at an ambient temperature of 22 ± 5°C and at 50–70% humidity. A 12-h day-night cycle was maintained to avoid fluctuations of the circadian rhythm within the rats and the rats were kept in translucent plastic cages with wood shavings provided as bedding. The cages were replaced with bedding before fasted rat testing, to prevent and lessen coprophagy. Rats were provided with *ad libitum* diet pellets (a nutrient composition of 38.5% fiber, 36.2% carbohydrate, 20.9% protein, and 4.4% fat, with a metabolizable energy content of 1.18 MJ/100 g (282 kcal/100 g)) and filtered drinking water throughout the experiment. During tests that required fasting, only water was supplied.

### Diabetes induction

A single intraperitoneal injection of 90 mg/kg, b/w of streptozotocin was administered to 48-h old rats (average weight 7 g; 40 rats of both sex) to induce type 2 diabetes [28]. The experiments were carried out 3 months after the injection of streptozotocin. Rats with a blood glucose level of 8–9 mmol/l at a fasted condition and >10 mmol/l at a postprandial state were selected as a type 2 diabetic model for the following experiments.

### Effects of *N. sativa* on glucose tolerance

Glucose (2.5 g/kg, b/w) was orally administered with or without plant extract (500 mg/kg, b/w) to 12 hr fasted type 2 diabetes rats (*n*=8). The blood was extracted and sampled from the tip of the tail before and after extract ingestion within time periods of 0, 30, 60, 90, and 120 min. The blood glucose was measured using an Ascencia Contour Blood Glucose Meter (Bayer, Newbury, U.K.).

### Effects of *N. sativa* on residual gut sucrose content

Glucose absorption was determined by the method as described by Hannan et al. [29]. Twenty-four hour fasted type 2 diabetic rats were provided with sucrose (2.5 g/kg of body mass) orally, with or without plant extract (0.5 g/kg, b/w). Following sucrose administration, rats were killed at 30, 60, 120, and 240 min, respectively, to measure the malabsorption of sucrose contents from six different parts of the GI tract. The GI tract was excised and six different segments including the stomach, the upper 20 cm, middle and lower 20 cm of the small intestine, the cecum and the large intestine were separated. Each segment was rinsed with acidified, ice-cold, saline and then centrifuged at 3000 rpm (1000×***g***) for 10 min. The supernatant was pipetted off and boiled for 2 h in H_2_SO_4_ to hydrolyze the sucrose content. The acid residue was then neutralized by 1 M NaOH solution. Both the plasma glucose concentration and the amount of glucose released from residual sucrose in the GI tract was determined. The GI sucrose content was calculated from the amount of liberated glucose [25].

### Effects of methanol extract of *N. sativa* intestinal glucose absorption

An *in situ* intestinal perfusion technique [30] was implemented for the estimation of intestinal glucose absorption impeding the effect of *N. sativa* in normal rats. Subjected animals were fasted for 36 h and anesthetized with sodium pentobarbital (50 g/kg, b/w) solution. The extract of *N. sativa* (10 mg/ml), equivalent to 0.5 g/kg, made up in Krebs–Ringer buffer containing glucose (54 g/l). The solution was passed through the pylorus, and the perfusate was collected at the ileum from a catheter inserted at the end. The control group received only Krebs’ solution containing glucose. Perfusion was carried out at a constant rate of 0.5 ml/min for 30 min at a maintained temperature of 37°C. Results were calculated as a percentage of absorbed glucose, of the glucose within the solution before and after the perfusion.

### Effects of *N. sativa* on gut motility

GI motility was determined using BaSO_4_ as described previously by Azad et al. [25]. The treatment group received the extract 1 h before consuming 10% BaSO_4_ (W/V of 0.5% Na-CMC). After providing BaSO_4_, animals were killed at 15 min after its administration and the distance travelled by BaSO_4_ was measured and calculated as a percentage of a total length of the small intestine (from the pylorus to ileocecal junction).

### Effects of *N. sativa* on intestinal disaccharidase enzyme activity

This experiment was carried out as previously described by Hannan et al. [29]. In brief, non-diabetic rats were fasted for 20 h, and then the methanol extract of *N. sativa* (500 mg/kg, b/w) was introduced by gastric gavage. The rats were then killed at 60 min after gavaging and the small intestine was extracted and cut lengthwise, bathed in ice-cold saline, and then homogenized in 10 ml of saline (0.9% NaCl).

The aliquots of homogenate were incubated at 37°C within a 40 mM sucrose solution for 60 min. A DCTM Protein Kit (Bio-Rad, U.S.A.) was used to measure the protein load. The concentration of sucrose transformation into glucose, represents the disaccharidase enzyme activity and was obtained as μmol/mg protein/h.

### Effects of *N. sativa* on insulin secretion from isolated islets

On the day of the experiment, islets were isolated and harvested from Long–Evans rats (180–250 g) pancreata using a collagenase digestion as described by Hannan et al. [31]. After preincubating the islets in KRB buffer containing 3 mM glucose for 40 min, islets (in groups of 8–10) were incubated for 1 h at 37°C in 500 μl KRB buffer containing 3 and 11 mM glucose along with methanol extract of *N. sativa* (Table 1). Aliquots of the resulting supernatant were stored at −20°C for insulin assay analysis using Rat Insulin ELISA Kit (Crystal Chem, U.S.A.).

**Table 1:** Effects of methanol extract of *N. sativa* on insulin secretion from isolated rat islets

Groups	Insulin secretion (ng/mg islet protein)	
	3 mM Glucose	11 mM Glucose
Control (Glucose alone)	2.23 ± 0.65	5.31 ± 0.74
*N. sativa* (25 μg/ml)	2.61 ± 0.33	6.37 ± 0.47*
*N. sativa* (50 μg/ml)	3.67 ± 0.33*	6.91 ± 0.33*
*N. sativa* (100 μg/ml)	5.87 ± 0.45^†^	8.49 ± 0.85*
*N. sativa* (200 μg/ml)	6.77 ± 0.35^†^	9.97 ± 0.45^†^
Glibenclamide (10 μg/ml)	7.55 ± 0.25^‡^	10.31 ± 0.65^†^

Isolated rat islets were incubated for 60 min with methanol extract of *N. sativa* (25–200 μg/ml) in the presence of 3 or 11 mM glucose; whereas Glibenclamide (10 μg/ml) used as a reference control, respectively. Values are Mean ± SEM with *n*=4. *P<0.05. ^†^*P*<0.01. ^‡^*P*<0.001 compared with control (3 and 11 mM glucose alone).

### Statistical analysis

All data were presented as mean ± standard deviation and statistical analysis was prepared on GraphPad Prism v5.0. A one-way ANOVA was carried out with a non-parametric Dunnett’s test for adjustment and interpretation; *P*<0.05 was considered as the minimum level of significance.

## Results

### Effects of *N. sativa* powder on *in vitro* glucose-adsorption capacity

**Figure 1:**
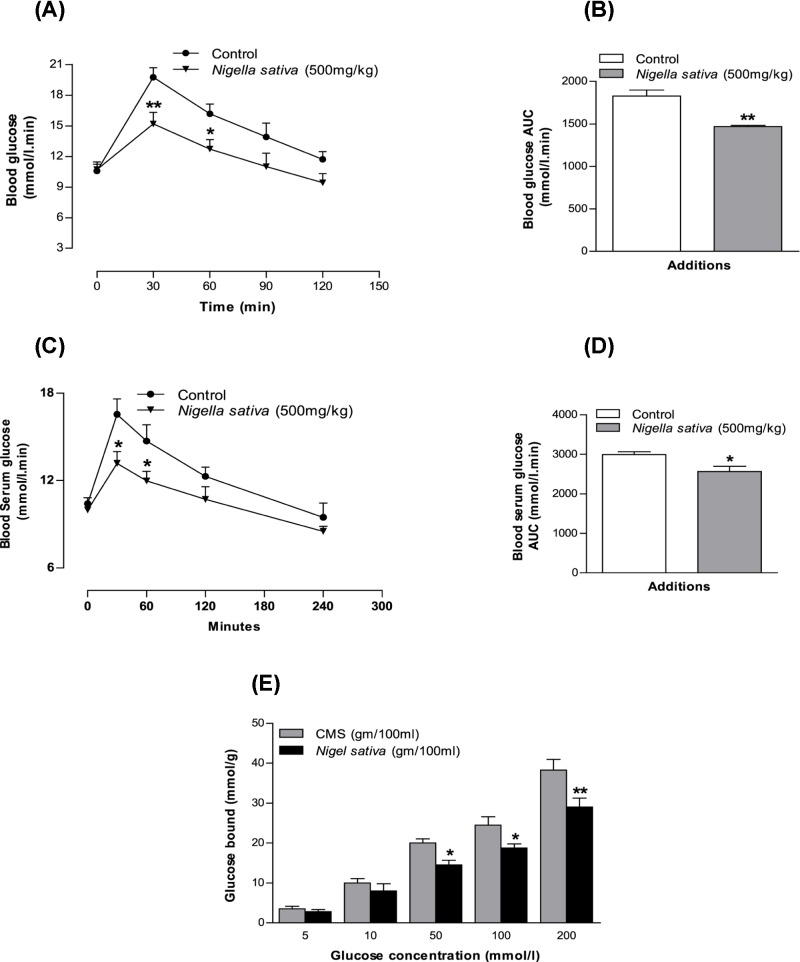
Effects of methanol extract of *N. sativa* on (A,B) glucose tolerance (GTT), (C,D) serum glucose after sucrose load (SGASL) in type 2 diabetic rats and (E) glucose adsorption capacity (GAC) *in vitro* Rats were fasted for 12 and 24 h and administered glucose or sucrose solution (2.5 g/kg, body weight) by oral gavage in presence or absence of methanol extract of *N. sativa* (500 mg/kg, body weight). Values are means and standard deviations represented by vertical bars (*n*=6, for GTT and SGASL and *n*=4 for GAC. The mean values that are marked with an asterisk (*) were substantially different from those of respective type 2 diabetic control rats (*P<0.05 and **P<0.01) alone (this was derived from repeated-measures ANOVA and adjusted using Bonferroni correction).

*N. sativa* powder illustrated the capacity of glucose adsorption at different concentrations of glucose within the solutions. This activity of adsorption continued from high concentrations of glucose to low concentrations of glucose (*P*<0.05, *P*<0.01; Figure 1E).

### Effect of *N. sativa* on glucose tolerance

Figures 1A,B show the effects of *N. sativa* extracts (500 mg/kg, body weight) on glucose tolerance. A significant decline was noted (*P*<0.05 and *P*<0.01) in the blood glucose concentration at 30 and 60 min following glucose ingestion (2.5g/kg, b/w). Type 2 diabetic rats that received *N. sativa* treatment had efficiently lowered glucose levels in comparison with rats that received glucose alone (*P*<0.05 and *P*<0.01; Figure 1A,B).

### Effects of *N. sativa* on serum glucose after the sucrose load

The glucose level of type 2 diabetic rats reached a peak 30 min after sucrose ingestion (Figure 1C,D). This rise in blood glucose due to sucrose load was suppressed by the methanol extract efficiently at both 30 min (*P*<0.05) and 60 min (*P*<0.05) in type 2 diabetic rats. This result may be a reflection of extracts activity on insulin secretion/action but also evidence of delaying the absorption of sucrose in the gut.

### Effects of *N. sativa* on unabsorbed sucrose content in the gut

**Figure 2:**
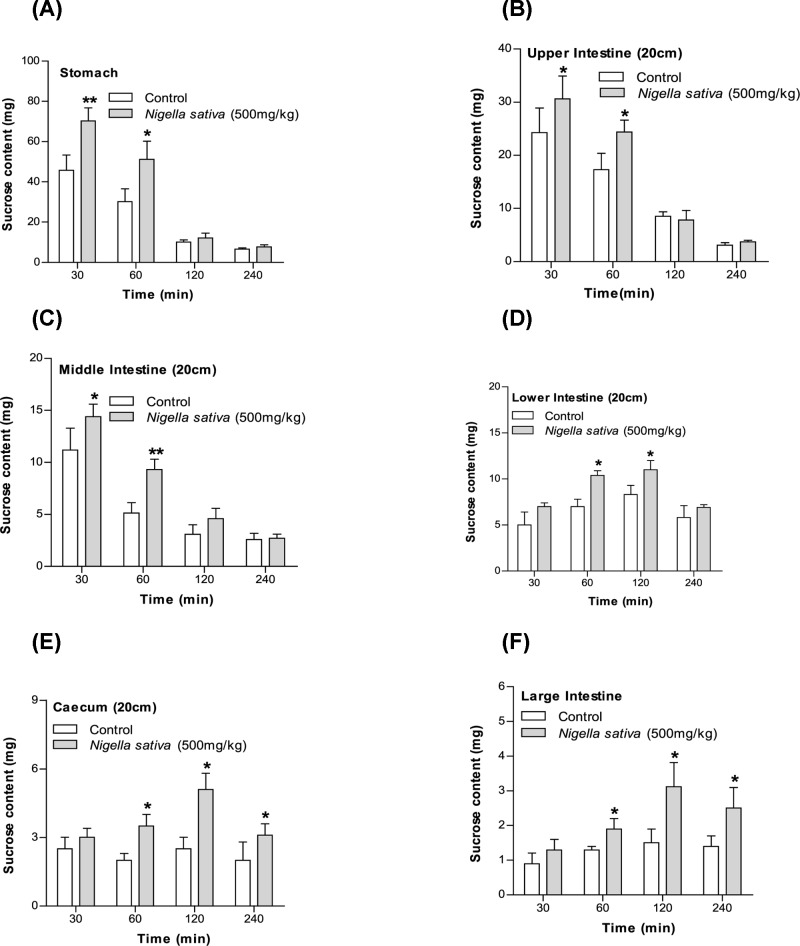
Effects of methanol extract of *N. sativa* on (A–F) GI sucrose content after oral sucrose loading in type 2 diabetic rats Type 2 diabetic rats were fasted for 24 h prior to the oral administration of sucrose solution (2.5 g/kg body weight) in the presence (treated group) or absence of (control group) methanol extract of *N. sativa* (500 mg/kg body weight). The values are means and standard deviations represented by vertical bars (*n*=6). The mean values that are marked with an asterisk (*) were substantially different from those of respective type 2 diabetic control rats (*P<0.05 and **P<0.01) (this was derived from repeated-measures ANOVA and adjusted using Bonferroni correction).

The unabsorbed sucrose content after the administration of sucrose (2.5g/kg, b/w) with methanol extract (500mg/kg, body weight) had increased significantly (*P*<0.05 and *P*<0.01; Figure 2) in the (A) stomach, (B) upper and (C) middle intestine after 30 min, in the whole small intestine after 1 h, and in the (D) lower intestine, (E) cecum, and (F) large intestine after 2 h. After 4 h, the sucrose content was detected in trace amounts in the control group, however, at the same time, sucrose was detected in the cecum as well as in the large intestine in the rat group that received the extract treatment (Figure 2).

### Effects of *N. sativa* on intestinal glucose absorption

**Figure 3:**
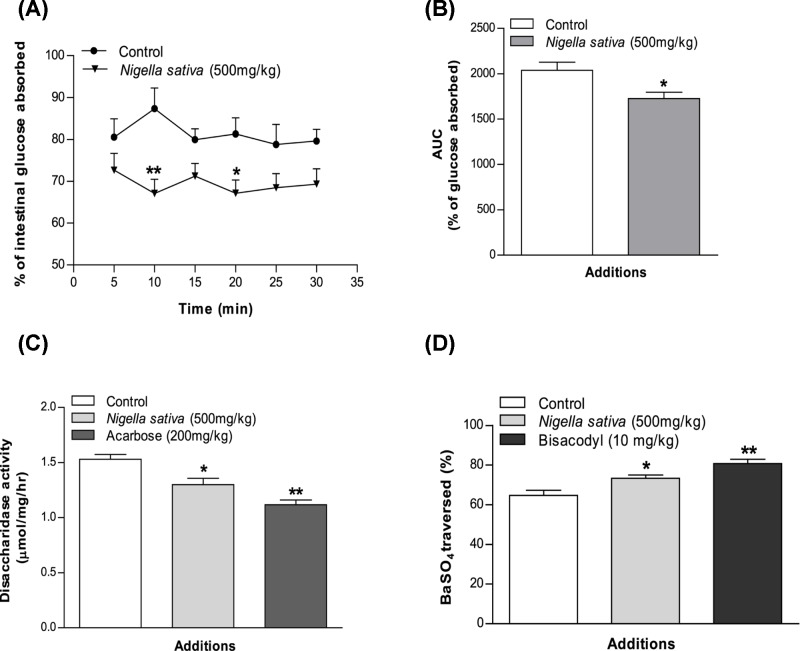
Effects of methanol extract of *N. sativa* on (A,B) intestinal glucose absorption, (C) disaccharidase enzyme activity, and (D) GI motility (by BaSO4 traversed) in non-diabetic rats Rats were fasted for 36 h (gut perfusion) and 20 h (enzyme activity and gut motility), and intestine was perfused with glucose (54 g/l) in the presence (treated group) or absence of (control group) methanol extract of *N. sativa* (10 mg/ml; with every individual obtaining 15 ml of perfusion). Enzyme activity was determined and BaSO_4_ administered at 60 min. Motility was measured over the following 15 min. Acarbose (ACB) (200 mg/kg) and bisacodyl (10 mg/kg) were used as standard drugs for disaccharidase activity and GI motility test correspondingly. The values are means and standard deviations represented by vertical bars (*n*=8). The mean values that are marked with an asterisk (*) were substantially different from those of respective control rats (*P<0.05 and **P<0.01) (this was derived from repeated-measures ANOVA and adjusted using Bonferroni correction).

Intestinal glucose absorption was almost constant during the 30-min period of perfusion with glucose. The glucose solution containing the extract decreased intestinal glucose absorption significantly (*P*<0.05 to *P*<0.01) at both 10 and 20 min of the perfusion period (Figure 3A,B).

### Effects of *N. sativa* on intestinal disaccharidase activity and GI motility

The methanol extract of *N. sativa* inhibited disaccharidase enzyme activity significantly (*P*<0.05, Figure 3C) in normal rats. Additionally, the extract showed potential to increase GI motility at a dose of 500 mg/kg, body weight (*P*<0.05, Figure 3D).

### Effects of *N. sativa* on insulin secretion from isolated islets

*N. sativa* extract effects on insulin secretion has been presented in Table 1. Extracts effect were assayed using isolated rat islets and compared in presence of 3 and 11 mM glucose. Extract concentration increases of 25–200 μg/ml had also increased the secretion of glucose-inducing insulin by 1.3–3 times in comparison with 3 and 11 mM glucose alone (*P*<0.01–0.001, Table 1). While, the dose-dependent increment in insulin release was 14–67% by the methanol extract in comparison with the 3 mM glucose alone (*P*<0.05 and *P*<0.01; Table 1), which has been increased to 2.3-fold with the increase in glucose concentration (11 mM). Whereas a positive control, Glibenclamide induced the insulin release from 1.9 to 3.4-folds in presence of 3 and 11 mM glucose.

## Discussion

The current study at hand used a streptozotocin-induced type 2 diabetes animal model; streptozotocin causes DNA damage and generates superoxide radicals to destroy the pancreatic β-cells [26]. *N. sativa* has established background as a potential antioxidant and anti-diabetic natural product due to its alkaloid derivatives, mostly thymohydroquinone and TQ [32,33]. It has been reported several times that TQ can protect β-cells from ROS damage and alleviate DM [34–37]. It was also indicated that supplementation with TQ (20 mg/kg, body weight/d, p.o.) during the gestation and lactation periods of diabetic mice protected their offspring from diabetes and its associated complications via decreasing the levels of blood glucose [38,39].

Hyperglycemia causes cellular damage that hinders the homeostatic regulation of internal glucose concentration, resulting in acutely altered cellular metabolism and long-term changes in cellular macromolecular content [40,41]. A postprandial glucose spike causes perturbation in endothelial cell function [42,43], and increases the risk of blood coagulation [43]. Hyperglycemic state also increases products of glycosylation, which in turn has a significant influence on the development of diabetes-induced vascular disease [44]. Consequently, management of hyperglycemic states is an essential method of diabetes control. There are some primary pathways used in anti-diabetic drugs that include enhanced insulin secretion, enhanced sensitivity to insulin, improved peripheral glucose utilization, inhibition of glucose absorption, and inhibition of carbohydrate digestion [45]. *N. sativa* presented promising glucose-lowering effect in the recent study with chemically induced diabetic rats. The methanol extract showed high efficiency in stimulating insulin secretion from the isolated rat islets. This effect was enhanced further with the increase in glucose concentration from 3 to 11 mM. Thus, the extract is increasing glucose sensitivity that leads into the increased insulin release and causes hypoglycemia [46].

An *in situ* intestinal perfusion of the GI tract showed a marked reduction in glucose absorption. Within the GI motility assay using BaSO_4_ milk, the intestinal motility was enhanced significantly by the methanolic seed extract. The Six Segment study of the GI tract accounted for a high amount of unabsorbed sucrose contents in the stomach, upper, middle, and lower intestines in groups that received extracts. The last three parts of the GI tract are the most important for the absorption of nutrients, including sugars [47]. *N. sativa* ability to slow sucrose absorption widely across the GI tract has been shown by the high amount of unabsorbed sucrose content left in the GI tract. Resulting from this, a substantial concentration of sucrose reached the large intestine and cecum, and remained unabsorbed and egested with feces.

A significant reduction in hyperglycemia after an oral sucrose load and an increased level of residual sucrose content throughout the gut was observed, including the critical last three parts of the GI tract. Disaccharides are not absorbed from gut unless converted into monosaccharides due to structural complexity. A high level of unabsorbed sucrose in the GI tract is a clear indication of reduced sucrose digestion. This decrement is further justified by the study with intestinal disaccharidase activity where the similar low-level absorption of sucrose was observed and this indicates a partial inhibition of the intestinal disaccharidase enzyme activity.

*N. sativa* seed extract showed a promising decline regarding disaccharidase enzyme activity. As complex carbohydrates require this enzyme to breakdown into simpler monosaccharides before absorption, any inhibition of this enzyme would interfere with sugar absorption, lowering the glycemic peak. In addition, the methanol extracts also showed improvement in glucose sensitivity and insulin release. However, precise mechanism of this inhibitory action remains to be studied.

## Conclusion

To conclude, the present study has shown that the effects of *N. sativa* seeds extract on anti-hyperglycemic activities in normoglycemic and diabetic animals are associated with a decreased intestinal glucose absorption and enhanced tissue glucose utilization that is mediated by the improvement in insulin release from islet. Also, a sound scientific basis appears to exist for the use of *N. sativa* as a dietary adjuvant for type 2 diabetes. Altogether, these findings suggesting a new mode of action of *N. sativa* in the treatment of DM that is co-related with its previous claims.

## Abbreviations

Aβ: Amyloid β
AD: Alzheimer’s Disease
b/w: Body Weight
DM: Diabetes mellitus
GAC: Glucose adsorption capacity
GI: Gastrointestinal
GOD-PAP: Glucose oxidase-phenol amino phenazone
KRB: Krebs Ringer Bicarbonate
OGTT: Oral glucose tolerance test
p.o.: Oral gavage
ROS: Reactive oxygen species
TQ: Thymoquinone
